# DNA methylation mediated genetic risk in severe acne in a young men population

**DOI:** 10.3389/fmed.2023.1196149

**Published:** 2023-07-24

**Authors:** Yujia Wu, Yun Chen, Bo Chen, Wenjuan Wu, Jiankang Yang

**Affiliations:** ^1^School of Basic Medical Sciences, Dali University, Dali, China; ^2^Department of Dermatology, First Affiliated Hospital of Kunming Medical University, Kunming, China

**Keywords:** severe acne, SNP, DNA methylation, genetic risk, methQTL

## Abstract

**Background:**

Acne is a chronic inflammatory skin disease that affects the pilosebaceous follicle and is influenced by heredity, hormones, inflammation, and the environment. At present, the recognized pathogenesis mainly includes four categories: excessive sebum secretion, excessive *Cutibacterium acnes* proliferation, excessive keratinization of sebaceous glands in hair follicles, and inflammatory mechanisms. Previous studies have found that DNA methylation is closely related to some chronic inflammatory skin diseases, and there is evidence that DNA methylation is controlled by genetic factors, making us want to know the relationship between DNA methylation, genetic variation and acne.

**Materials and methods:**

In our previous study, we performed genome-wide DNA methylation analysis in peripheral blood samples from 44 patients with severe acne and 44 unaffected normal subjects, and identified 23 differentially methylated probes (DMPs). In this study, we identified single nucleotide polymorphisms (SNPs) associated with severe acne by genome-wide association analysis in these 88 samples. To test the association between SNPs and DMPs, we conducted DNA methylation quantitative trait loci (methQTL) analysis. Next, causal inference testing (CIT) was used to determine whether genetic variation influences DNA methylation, which impacts disease phenotypes.

**Result:**

We found 38,269 SNPs associated with severe acne. By methQTL analysis, we obtained 24 SNP-CpG pairs that reached the threshold (FDR < 0.05), which included 7 unique CpGs and 22 unique methQTL SNPs. After CIT analysis, we found that 11 out of 24 pairs of SNP-CpG showed a weakened SNP effect after adjustment for methylation, indicating a methylation-mediated relationship between SNPs and severe acne. These 11 SNP-CpG pairs consist of four unique CpG sites and 11 SNPs, of which three CpG sites, cg03020863, cg20652636, and cg19964325, are located on the gene body of PDGFD, the intron of SH2D6, and the 5’UTR of the IL1R1 gene, respectively.

**Conclusion:**

During this study, the DNA methylation of certain genes was found to be influenced by genetic factors and mediated the risk of severe acne in a young Chinese male population, providing a new perspective on the pathogenesis of severe acne.

## 1. Introduction

One of the most common diseases of the skin worldwide, acne is an inflammatory disease of the pilosebaceous follicle that contributes to a prevalence rate of up to 85% in adolescents and young adults (1). Acne has not been fully explained by its pathogenesis, but there are four recognized factors: excessive sebum production, hyperproliferation of *Cutibacterium acnes*, hyperkeratosis of the follicular sebaceous glands, and inflammatory mechanisms. In some patients, the onset of acne is also influenced by genetic, immune, endocrine, emotional, and dietary factors.

Acne can be classified as mild, moderate, or severe based on the severity of the clinical lesion (corresponding to grade IV in the Pillsbury classification) (2). Severe acne is characterized by acne, papules, and pustules all over the face, neck, chest, and back, as well as a heavy inflammatory response, nodules and cysts, and scarring. Severe acne can cause discomfort, emotional stress, and even permanent scarring on the skin, which can lead to cosmetic anxiety and low self-esteem, seriously affecting the patient’s quality of life (3). Combined with its high recurrence rate, we believe it is important to investigate the genetic and regulatory mechanisms associated with the development of severe acne.

Single nucleotide polymorphisms (SNPs) result from single nucleotide variation, which refers to sequence polymorphism at the genomic level, including single-base deletion, insertion, transition, and transversion. There is usually a single nucleotide polymorphism between C and T, because cytosines in GC-rich regions are typically methylated and methylated cytosines are transferred to thymine following spontaneous deamination. In terms of their location in the genome, SNPs can be categorized into three categories: gene-coding SNPs, intergenic SNPs, and perigenic SNPs (4), and genetic diseases are closely linked to them. The emergence of SNPs in genes or specific regulatory regions may have a direct effect on gene function, and in recent years, SNPs have been widely used as markers for the development of some cancers and genetic diseases (5).

DNA methylation (DNAm), a relatively stable state of modification, is one of the epigenetic regulatory mechanisms that reversibly modifies cytosines in the human or other eukaryotic genomes to 5-methylcytosine. DNA Methyltransferase (DNMT) has been shown to play a significant role in epigenetics and to be closely related to gene regulation (6). Interaction between DNA methylation and transcription factor regulated gene expression (7). DNA methylation normally rich in CpG islands will block transcription factor (TF) binding and result in gene silencing. In contrast, hypomethylation of CpG islands (CGI) in the promoter region results in active transcriptional gene expression. However, some studies suggest that DNA methylation may not cause binding to transcription factors or transcriptional activity, but rather may result from it (8). The DNAm regulates gene expression, thereby affecting biological processes. It is also possible to modulate DNAm through genetics (SNPs), potentially mediating genetic risks (9). These SNPs are called methylation quantitative trait loci (methQTL) and can influence the methylation pattern. As a result, disease-associated DNAm differences may result from a disease or may be driven independently by a genotype. A recent study has demonstrated the significant role of methylation quantitative trait loci (methQTL) in psoriasis (10).

DNA methylation has been widely used in the study of the pathogenesis of cancer and some common inflammatory diseases (11, 12). Although there are still few studies on DNA methylation associated with severe acne, several inflammatory skin diseases, including psoriasis and systemic lupus erythematosus, have been found to be associated with DNA methylation, from which we infer that DNA methylation may play an influential role in the development of severe acne (13, 14). A previous study identified 23 differentially methylated probes (DMPs) associated with severe acne (15). However, the potential mechanism by which these methylation markers are associated with severe acne is not clear. We hypothesized that DNA methylation might mediate the genetic risk of severe acne. The purpose of this study was to explore the role of genetic and epigenetic variation in severe acne by integrating SNPs, DNAm, and acne phenotype. We used blood-derived genetic and epigenetic data from 44 severe acne patients and 44 normal controls to associate acne-related SNPs with acne-related methylation sites using methQTL and evaluate the potential mediating effect of methylation sites on acne-related SNPs using CIT analysis. The final determined methylation site serves as a mediator, receiving regulation from SNP sites and influencing disease phenotypes. These methylation sites, which play a mediating role, provide new ideas for further exploring the pathogenesis of acne.

## 2. Materials and methods

### 2.1. Sample source

There were 88 samples, 44 controls and 44 severe acne patients each. Healthy controls and patients with severe acne were all Chinese Han Chinese unrelated to each other. To reduce the effect of age and sex on methylation levels, we restricted all samples to male blood samples and all were between the ages of 18–26 years (Supplementary Table S1).

### 2.2. Genome-wide genotyping and quality control

Genome-wide genotyping analysis of the 88 samples was performed using the Illumina HumanOmniZhongHua-8v1.4 BeadChip, which detects 900,000 SNPs. Genotype dataset has been deposited in the OMIX databases (OMIX705). SNP loci were then quality-controlled: (i) to exclude SNP loci with a detection rate of <98% in all samples; (ii) to exclude SNP loci with a minimum allele frequency (MAF) <0.05 in all samples; and (iii) to exclude SNP loci in healthy control samples that deviated significantly from Hardy–Weinberg equilibrium (*p* < 1 × 10^–4^). After quality control, a total of 809,305 SNP loci were screened. SNPs were found to be associated with severe acne by genome-wide association analysis (FDR < 0.05) with plink software.

### 2.3. Data of differential methylation sites

We used methylation dataset that we had previously stored in the OMIX databases (OMIX704). The dataset includes genomic methylation levels of 88 samples, which are the same samples as those used for genome-wide genotyping analysis. They were detected using the Illumina Infinium Methylation EPIC BeadChip, which can quantitatively measure more than 865,911 methylation sites. We described the methylation assay for each sample in a previous study (15), from which we obtained 23 differentially methylated loci significantly associated with severe acne (Supplementary Table S2).

### 2.4. Identification of methylation quantitative trait loci

The relationship between 23 differentially methylated CpG loci associated with severe acne and genome-wide associated SNPs associated with severe acne were examined by methQTL analysis. Because the sample selected was all male, the influence of gender on the study was reduced. We used linear additive regression models with Matrix eQTL software (16) for association analysis. In order to perform multiple comparison correction, we used milder Benjamini–Hochberg corrections. In this study, *cis*-MethQTL was defined as less than 500 kb upstream and downstream of CpG sites on the same chromosome, while trans-MethQTL was defined as greater than 500 kb from the target CpG site on the same chromosome or different chromosomes.

### 2.5. Causal reasoning test

**Figure 1:**
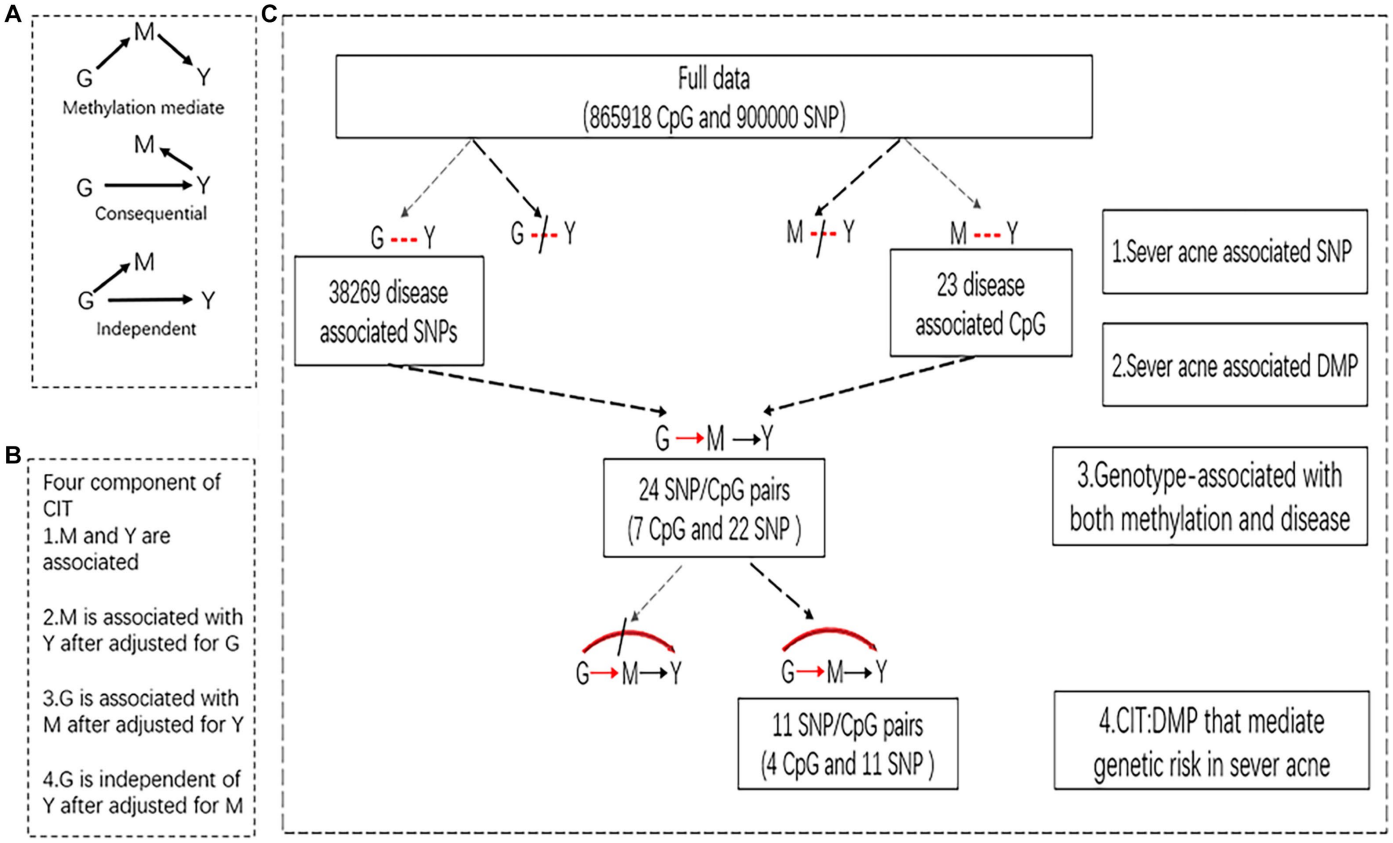
Identification of epigenetically mediated genetic risk factors for severe acne. **(A)** Possible relationship between a causal factor (G), a possible mediator (M), and an outcome (Y). Top, is the methylation-mediated relationship, in which genotype (G) acts on phenotype (Y) through methylation (M); middle, is the consequential methylation model, in which DNA methylation (M) changes are the consequence of phenotype (Y); bottom, the independent model, in which the genotype (G) acts on DNA methylation (M) and phenotype (Y) independently. **(B)** The four components of the CIT. **(C)** Flow diagram and results for identifying epigenetically mediated genetic risk for severe acne.

Causal Inference Testing (CIT) can be used to test whether potential intermediate variables between processing variables and outcome variables affect results. In this study, the causal factors (genotype, G) used by CIT to evaluate the disease outcome (severe acne, Y) are mediated by a third mediator (DNA methylation, M). To clarify that methylation plays a mediating role in the genetics of severe acne, the following criteria should be met: (i) G and Y are associated, (ii) M is associated with Y by controlling for G, (iii) G is associated with M by controlling for Y, and (iv) G and Y are independent by controlling for M. If M is a consequence of Y or is independently controlled by G, then there should be no difference in the effect of G on Y when M is regulated. However, when M mediated the genetic risk of severe acne (Table 1), modulating M significantly reduced the effect of G on Y (Figure 1). Through CIT analysis, we focused on investigating whether methylation as a mediator is regulated by SNP sites and affects disease phenotype. The comprehensive value of *p* for CIT is the value of *p* calculated using the Intersection–Union Test framework, which is the upper bound of the four *p*-values for component testing (17). Testing in this study was performed using the “cit” package in R. The CIT significance threshold was set at *p* < 0.05.

**Table 1:** Methylation sites mediate genetic risks in acne.

Severe acne associated DMPs						SNPs associated with DMPs							
lllumina ID*	Gene_context	Gene name	DeltaBeta	*P-*value (Meth vs. Pheno)	FDR	SNP	Gene	chr	Adjusted *p*-value (Geno vs. Pheno)	Adjusted *p-*value (Meth vs. Pheno)	Adjusted *p-*value (Meth vs. Geno)	Adjusted independent *p-*value (Geno vs. Pheno)	CIT P^†^
cg13102742	IGR		−0.24	2.30E-04	1.02E-03	rs7215290	None	chr17	6.36E-03	2.34E-03	4.87E-03	0.008	0.00821
cg03020863	Body	PDGFD	−0.09	1.70E-08	6.82E-07	rs11226216	None	chr11	1.34E-02	1.67E-05	6.99E-04	0.000	0.01337
cg13102742	IGR		−0.24	2.30E-04	1.02E-03	rs17138064	None	chr17	4.58E-03	2.18E-03	1.40E-02	0.007	0.01397
cg03020863	Body	PDGFD	−0.09	1.70E-08	6.82E-07	rs17102225	None	chr11	8.87E-03	1.17E-05	2.57E-02	0.001	0.02568
cg20652636	IGR	SH2D6	0.07	3.12E-04	1.11E-03	rs7340340	ELMOD3	chr2	2.41E-02	2.58E-02	1.08E-04	0.017	0.02577
cg03020863	Body	PDGFD	−0.09	1.70E-08	6.82E-07	rs4754106	None	chr11	4.06E-03	3.38E-05	2.75E-02	0.002	0.02745
cg03020863	Body	PDGFD	−0.09	1.70E-08	6.82E-07	rs361296	PDGFD	chr11	2.86E-02	9.04E-06	1.24E-03	0.000	0.02863
cg20652636	IGR	SH2D6	0.07	3.12E-04	1.11E-03	rs2292653	TGOLN2	chr2	3.19E-02	3.13E-02	1.56E-07	0.014	0.03189
cg20652636	IGR	SH2D6	0.07	3.12E-04	1.11E-03	rs6886	CAPG	chr2	3.51E-02	2.16E-02	8.99E-05	0.025	0.03505
cg20652636	IGR	SH2D6	0.07	3.12E-04	1.11E-03	rs6727913	SH2D6	chr2	3.82E-02	3.15E-02	5.95E-11	0.015	0.03823
cg19964325	5’UTR	IL1R1	−0.06	2.98E-04	1.11E-03	rs12624116	IL1R1	chr2	3.92E-02	1.95E-02	2.37E-03	0.037	0.03919

Gene name, according to HUGO Gene Nomenclature Committee; Gene_context, location of the gene associated CpG-site(s) with respect to the gene context *Illumina ID is arranged by significance rank of CIT test. Adjusted P value (Meth vs Pheno): Controlling genotype, DNA methylation is associated with severe acne. Adjusted P value (Meth vs Geno): Control of severe acne, association of DNA methylation with genotype. Adjusted independent *p* value (Geno vs Pheon): Whether genotype and severe acne are independent of each other under the control of DNA methylation. ^†^CIT P, the maximum of the component *P*-values for an omnibus test.

## 3. Results

### 3.1. Selection of single nucleotide polymorphisms

**Figure 2:**
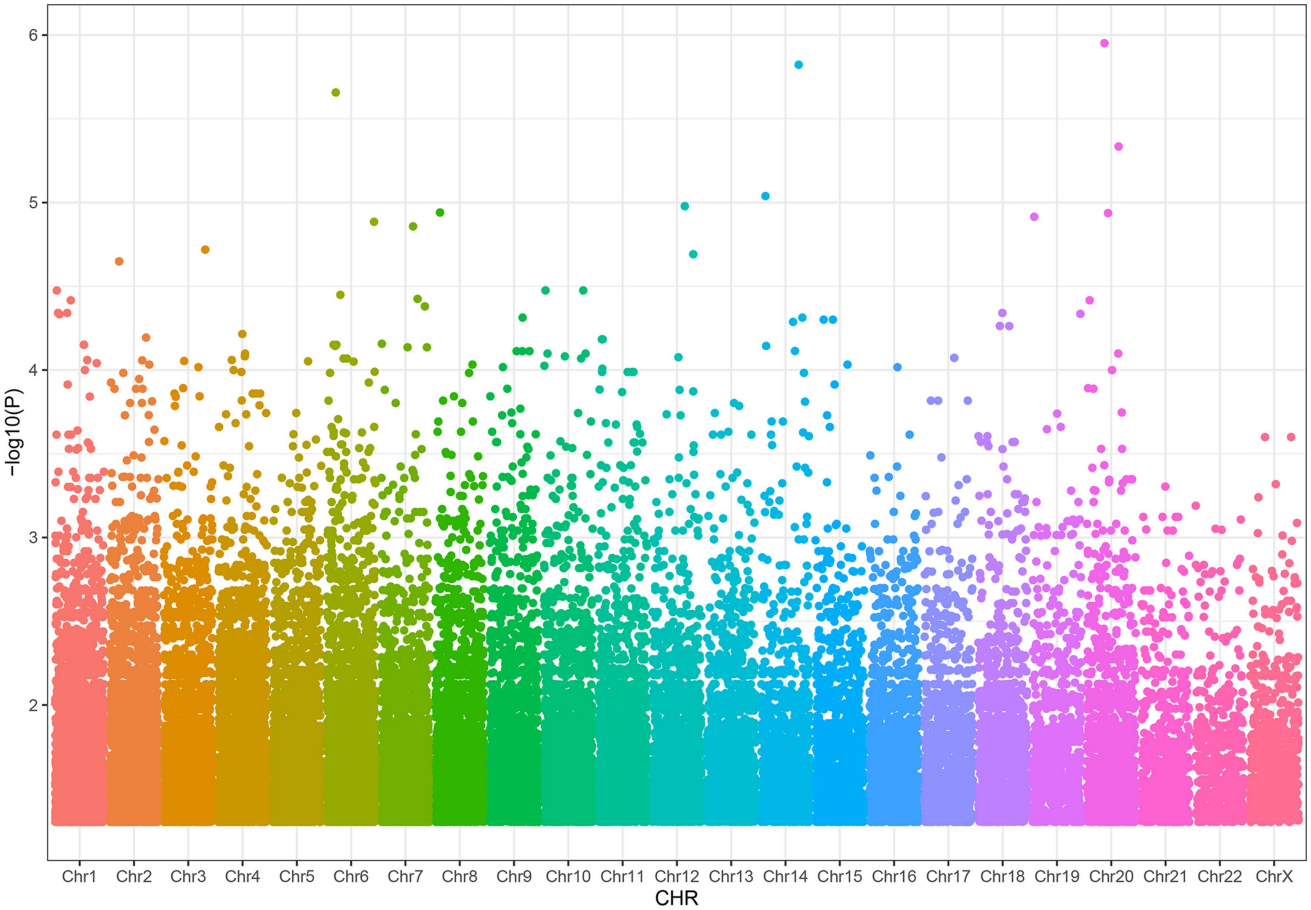
Distribution of SNPs associated with severe acne.

We found that 38,269 SNPs were linked to severe acne (*p* < 0.05), including 77 significant SNPs (*p* < 1 × 10^−4^, Figure 2). About 11% of the SNPs were located on chromosome 6 and 11% on chromosome 1. Approximately 66% of these SNPs resulted in intron variants in some genes, while approximately 24% of the SNPs are not located on genes.

### 3.2. Identification of methylation quantitative trait loci

**Figure 3:**
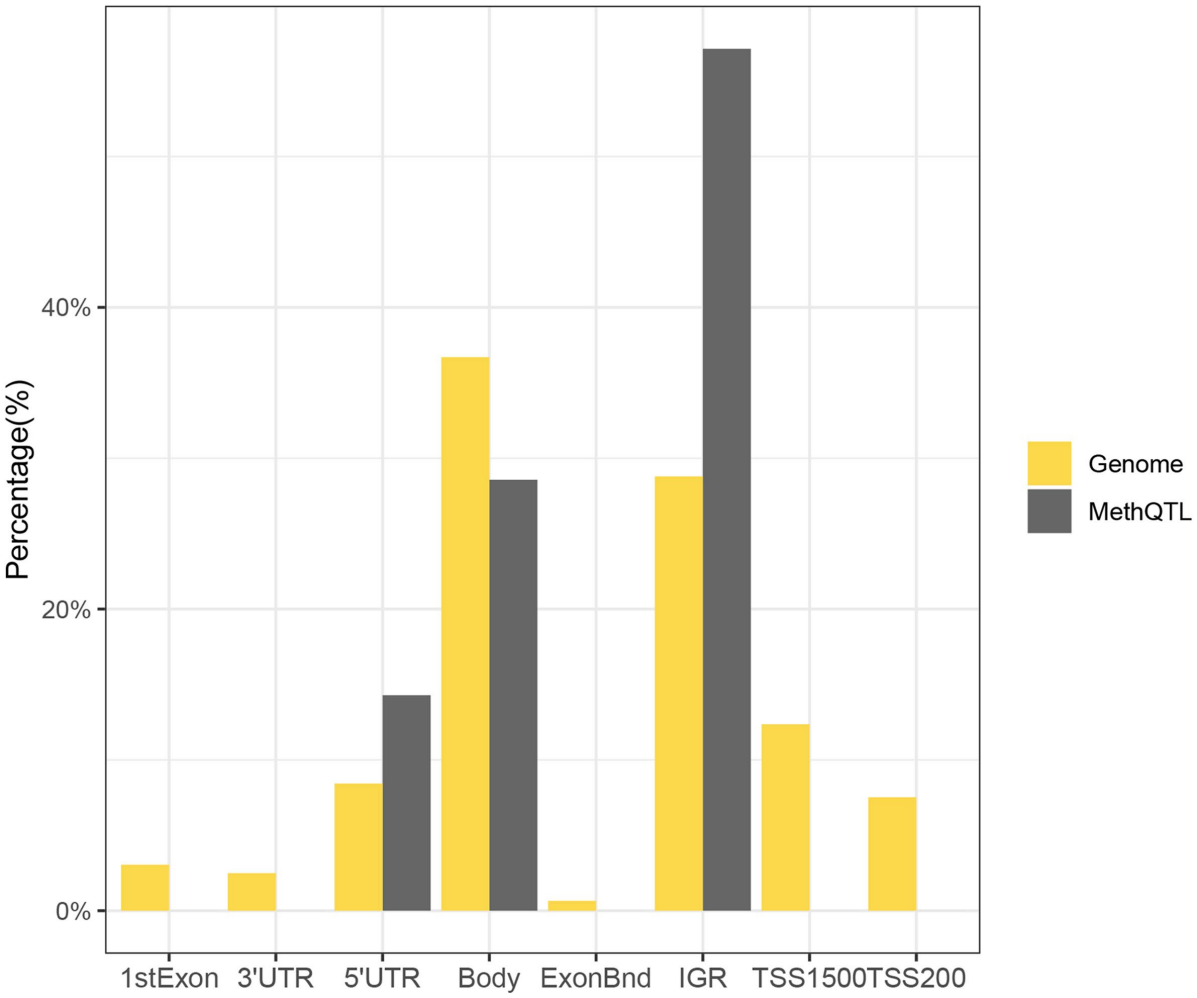
The methylation pattern of MethQTL CpG sites is described. Based on their positions in RefSeq genes, the distribution of CpG sites is determined. The term “Genome” encompasses all the methylation sites that have been mapped (*N* = 865,918). “MethQTL” denotes the methQTL CpG sites (*N* = 7) that are regulated by genetic markers. 1stExon (First Exon), first exonic region on the gene; 3’UTR, between the stop codon and poly A signal; 5’UTR, within the 5′ untranslated region, between the TSS and the ATG start site; Body, between the ATG and stop codon; irrespective of the presence of introns, exons, TSS, or promoters; ExonBnd, exon boundaries; IGR (Intergenic region), a DNA sequence between genes; TSS1500, 200–1,500 bases upstream of the transcriptional start site (TSS); TSS200, 0–200 bases upstream of the TSS.

We discovered that 24 SNP-CpG pairs attained a statistically significant threshold (Benjamini-Hochberg corrected FDR < 0.05), which included 7 unique CpG sites and 22 unique SNPs (Supplementary Table S3). The CpG probes for methylation quantitative trait loci (methQTL) exhibited differences in various annotated regions, with 57.14% (4/7) of CpG probes located in intergenic regions, 28.57% (2/7) of CpG probes located in gene bodies, and 14.29% (1/7) of probes located in 5 ‘UTR regions (Figure 3). No CpG probes were detected in the 1stExon region, ExonBnd region, TSS200 region, TSS1500 region and 3 ‘UTR region. The distribution of MethQTL CpG probes was observed to be higher in the Intergenic region and 5’UTR regions compared to the distribution of CpG loci across the genome. The intergenic and 5’UTR regions of CpG probes were found to have a higher likelihood of containing methQTL compared to the gene body, 1stExon region, ExonBnd region, TSS200 region, TSS1500 region, and 3’UTR region.

### 3.3. Setting up epigenetic mediation in genetic risk

**Figure 4:**
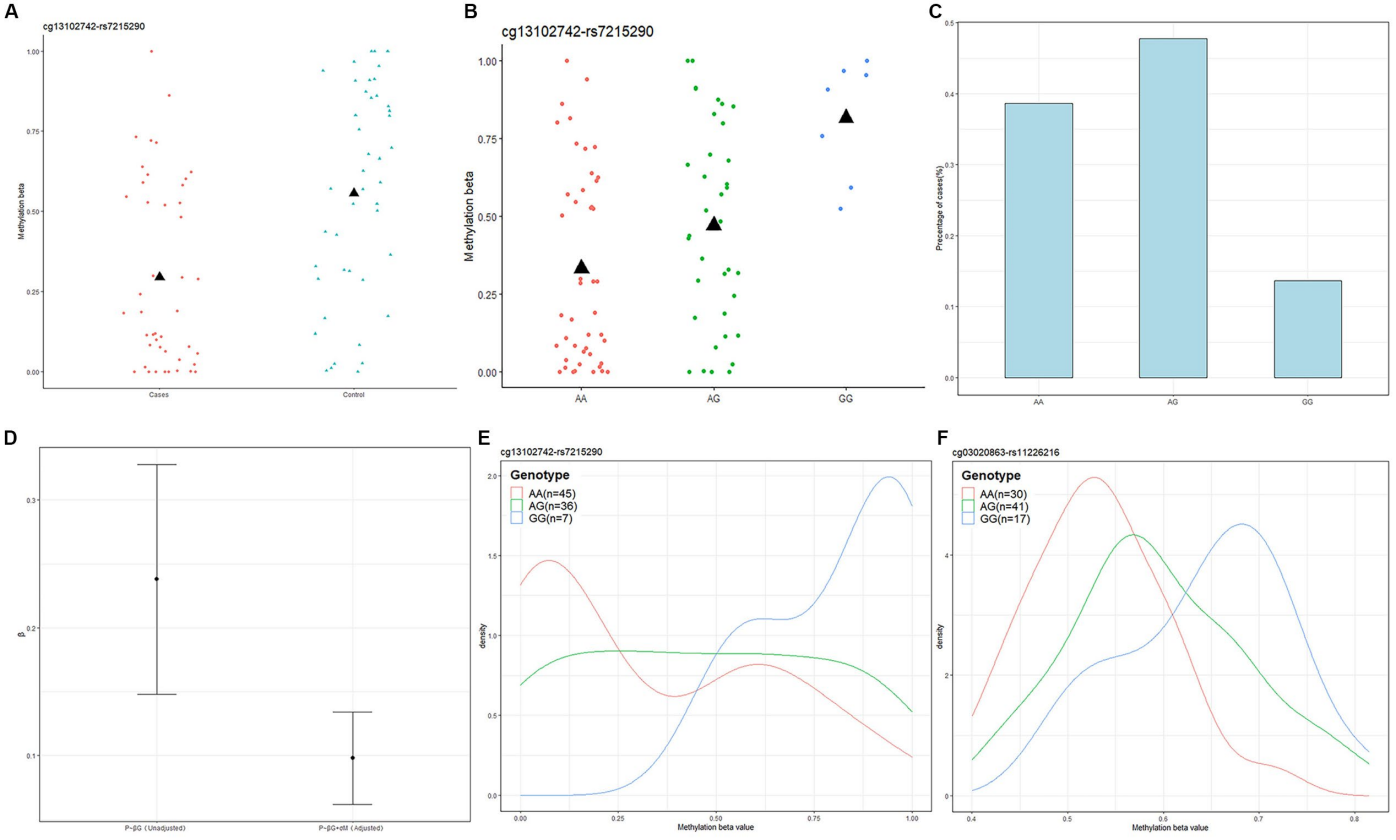
Genotype-dependent CpGs potentially mediate genetic risk for acne and the relationship among cg13102742, rs7215290, and severe acne. **(A)** The correlation between cg13102742 and the condition of the disease. **(B)** The correlation between cg13102742 and rs7215290. The black triangle symbol represents average DNA methylation levels. **(C)** Percentage of cases for each rs7215290 genotype. **(D)** The beta (β) value indicates the correlation between the severe acne phenotype (P) and genotype (G), with or without considering the DNA methylation level of cg13102742 (M). **(E,F)** Instances of DMPs that may potentially influence the genetic susceptibility to severe acne. Density plots displaying the methylation levels based on genotype for two SNP-CpG pairs. The lines on the plot were shaded with different colors to represent various genotypes.

Next, we used Causal Interference Testing (CIT), a hypothesis testing method based on likelihood. It adds a third potential mediating variable to two related variables and can calculate *p*-values for three variables to assess whether this potential mediator may mediate a known causal relationship between a genotype and disease phenotype. The primary aim of this investigation was to evaluate the potential mediation of SNPs by CpGs. After conducting CIT testing on these 24 pairs, we observed that the SNP effects of 11 pairs were attenuated after adjusting for methylation (Table 1). These 11 SNP-CpG pairs include 11 unique SNPs and 4 unique CpGs. The result demonstrates that the relationship between these 11 SNPs and severe acne is mediated by methylation. Among them, cg13102742 (*p* = 0.008214) had a remarkable mediating effect on rs7215290 (Figure 4). After adjusting for cg13102742, the effect of rs7215290 on severe acne decreased from 0.238 to 0.14.

## 4. Discussion

Acne is a chronic skin condition characterized by inflammation, and several factors may play a role in its onset. It is generally believed that *Cutibacterium acnes*, sebaceous glands, and follicular keratinocytes are involved in the development of acne. It has recently been suggested that staphylococci are also involved in this process and that the rate correlates positively with the severity of the disease (18). Patients with severe acne have been observed to have a significant ecological dysbiosis in their cutaneous microbiome (19). It has been shown that an excessive inflammatory response can lead to the development of severe acne. In the PAPA spectrum syndrome, severe acne may be the result of overexpression of its inflammatory component (20). Furthermore, the inflammatory process of acne is linked to cytokines such as TWEAK, IL-19, and IL-17, with IL-19 being linked to the severity of acne and suggested as a predictive inflammatory marker for typical acne (21, 22). The influence of genetic factors on the development of acne has also been demonstrated, but there is little research on the influence of epigenetic regulatory mechanisms on severe acne. In this study, therefore, we examined genotype and epigenetic data obtained from blood samples, primarily to search for methylation sites that could potentially influence the risk of developing severe acne.

We correlated disease-associated SNPs with differentially methylated loci and used methQTL analysis to find 24 SNP-CpG pairs that reached a significant correlation threshold, constituting 22 unique SNPs and 7 unique CpGs. methQTL CpG probes differed across annotation regions, with more than half located in the intergenic region, while those in the 1stExon region, the ExonBnd region, the 3′ UTR, the TSS1500 region, or the TSS200 region were absent. MethQTL CpG probes were enriched in the intergenic and 5′ UTR regions compared to the genome. Similar to the current study, in the identification of 55,000 repetitive DNA methylation QTLs, it was found that trans-methQTL DNA methylation sites were significantly increased in the 5′ UTR region (23). The 5′ untranslated region (UTR) plays a crucial role in regulating post-transcriptional control and translational efficiency. Additionally, the introns present in this region are abundant in genes that have regulatory functions (24). Recent research has demonstrated that not all significant methylation events take place in the gene’s promoter region. Methylation that is functionally important can also occur in the intergenic region (25). CpG dinucleotides’ cytosine residues are the most mutagenic sites in the human genome, and the majority of them are methylated. These residues can undergo spontaneous deamination to produce thymine. This mutation is affected by many factors. Some studies have discovered that the environment (chronic social pressure) influences the methylation pattern of an intergenic region of the X chromosome (26). In this study, the enrichment of the MethQTL CpG probe in the intergenic region can be explained by the fact that the emotions of anxiety and stress affect the methylation level between genes, or consider the methylation between genes induced by chronic inflammation in the organism.

An association between genetic variation, methylation levels, and disease phenotype has been noted in several studies, with both local (*cis*) and distal (*trans*) aspects of genetic variation being connected to methylation levels. As with the study on schizophrenia, all SNPs in this study were cis-MethQTL (27). Most methQTLs have been shown to act in cis rather than *trans* (23). At the same time, methQTL in our results also showed *cis*-like rather than trans effects. This phenomenon can be explained by the small sample size we selected and the significance threshold set for the analysis (*p* = 2.6 × 10^−7^). Therefore, we need more peripheral blood samples to assess trans-QTL.

By using the CIT test, we found 11 SNP-CpG pairs with four unique CpG sites mediating the relationship between the 11 SNPs and severe acne phenotype. We analyzed 11 SNP-CpG pairs (according to *P*_CIT_ < 0.01) and found four unique CpG sites, of which cg13102742 is located in the intergenic region (IGR) of chromosome 17, cg03020863 is located on the platelet-derived growth factor D (PDGFD) gene body, cg20652636 is located on the SH2D6 intron variant (intron variant), and cg19964325 is located in the 5‘ UTR of IL1R1. According to our findings, the most prominent cg13102742 is found in the IGR of chromosome 17, not on any gene. The CpG site cg03020863 is located on the PDGFD gene body, and PDGFD encodes a protein that is a member of the platelet growth factor (PDGF) family. PDGF is a significant growth factor that regulates cell growth and division. Studies have shown that PDGFD amplifies the activity of human natural killer (NK) cell effectors when it is combined with the NKp44 receptor. In addition, it can mediate IL-15-induced survival of human NK cells through binding to PDGFRβ (28). Natural killer (NK) cells are crucial immune cells that participate in various functions, including anti-tumor responses, antiviral infections, immune regulation, and, in some cases, the development of hypersensitivity and autoimmune diseases. A cardiovascular disease risk study found that PDGFD promotes proliferation, migration, and inflammatory factor expression in cardiovascular adventitial fibroblasts (29). Fibroblasts are involved in wound repair and play an important role in wound healing. Severe acne causes local skin damage, and fibroblasts are involved in this repair process, so we considered whether PDGFD also affects fibroblasts in the skin to participate in the development of inflammation and skin repair. Cg20652636 is situated on the intron variant of SH2D6, which could play a role in intracellular signal transduction and transmembrane receptor protein tyrosine kinase signaling pathways. A copy number variation analysis suggests that microdeletion of SH2D6 may be responsible for the generation of chimeric transcripts by ELMOD3 and SH2D6 fusions, which may be involved in autism spectrum disorder (ASD) phenotypes along with other variants (30). Some research suggests that daily stress, depression, and anxiety can contribute to the development of acne and can also increase the severity and duration of its symptoms (31). We also found that cg19964325, located in the 5’ UTR of the IL1R1 gene, could be a potential gene for the genetic risk of acne. IL1R1 is a cytokine receptor that encodes the interleukin-1 receptor family. The IL-1β-IL-1R signaling pathway was found to promote skin inflammation by amplifying the inflammatory cascade response, possibly by directly regulating skin IL-17 producing cells and stimulating keratin-forming cells to form cytosolic factors (32). In the extracellular space, IL-1α bound or released to membranes can bind to IL-1R1 on the cell surface, which also induces the production of inflammation (33). These studies suggest that IL1R1 is a key factor in the development of skin inflammation, and acne belongs to chronic inflammatory diseases of the skin. It has been shown that porphyrins produced by *Cutibacterium acnes* activate NLRP3 inflammatory vesicles and promote IL-1β secretion (34–36). Therefore, we consider that the IL-1β-IL-1R signaling pathway may emerge as a key player in the developmental mechanism of severe acne. While it remains to be investigated how epigenetic mechanisms mediate genes to influence disease development, current studies suggest that in the DNAm state, it is environmentally sensitive and can mediate phenotypic plasticity.

However, we must acknowledge that our findings are based on the limited sample data available and have certain limitations. These findings must be confirmed in a larger and independent sample size. Furthermore, the results obtained from the analysis are not entirely conclusive, and further functional experiments on the pathogenesis are still necessary to validate them. Nevertheless, these methylation markers can serve as a starting point for further research on severe acne.

## 5. Conclusion

This study is one of the few to assess the correlation between severe acne, methylation, and genotype in young men. We identified several methQTL CpG loci and SNPs associated with severe acne. The methQTL CpG loci were enriched in the Intergenic region. We found that four CpG loci mediated the genetic risk of severe acne, three of which, cg03020863, cg20652636, and cg19964325, were located on the PDGFD gene body, the SH2D6 intron variant, and the 5’ UTR of the IL1R1 gene, respectively. The most significant CpG locus, cg13102742, was not located on any gene. By performing methQTL analysis of GWAS SNP data and DNA methylation data, and assessing the mediating role of DNA methylation using CIT analysis, we identified three possible susceptibility genes for severe acne, providing new insights into the pathogenesis of severe acne.

## Data availability statement

The datasets presented in this study can be found in online repositories. The names of the repository/repositories and accession number(s) can be found in the article/Supplementary material.

## Ethics statement

The studies involving human participants were reviewed and approved by Medical Ethics Committee of Dali University. The patients/participants provided their written informed consent to participate in this study.

## Author contributions

YW studied the data and algorithm concepts, performed the data analysis and statistics, and drafted the manuscript. YC and BC analyzed the data. JY and WW designed the research and analysis and revised the manuscript. All authors contributed to the article and approved the submitted version.

## Funding

This study was supported by grants from the National Natural Science Foundation of China (81760565 and 82160600), Youth Top Talent project of High-level talent development support program of Yunnan Province (YNWR-QNBJ-2020-239), Li Yunqing expert workstation of Yunnan Province (202005AF150014), and Yunnan College Students’ Innovation Training Project (S202210679008).

## Conflict of interest

The authors declare that the research was conducted in the absence of any commercial or financial relationships that could be construed as a potential conflict of interest.

## Publisher’s note

All claims expressed in this article are solely those of the authors and do not necessarily represent those of their affiliated organizations, or those of the publisher, the editors and the reviewers. Any product that may be evaluated in this article, or claim that may be made by its manufacturer, is not guaranteed or endorsed by the publisher.
